# Bioaccumulation and Biomagnification of 2-Ethylhexyl-4-dimethylaminobenzoate in Aquatic Animals

**DOI:** 10.3390/ijerph15112395

**Published:** 2018-10-29

**Authors:** Guanghua Lu, Ranran Zhou, Sheng Li, Tianjian Dang, Jianchao Liu

**Affiliations:** 1 Water Conservancy Project and Civil Engineering College, Tibet Agriculture and Animal Husbandry University, Linzhi 860000, China; ghlu@hhu.edu.cn (G.L.); txdangtianjian@163.com (T.D.); 2 Key Laboratory for Integrated Regulation and Resources Development on Shallow Lakes of Ministry of Education, College of Environment, Hohai University, Nanjing 210098, China; zrr0416@hhu.edu.cn; 3 Jiangsu SinoRoad Engineering Research Institute Co., LTD, Nanjing 211806, China; env_ls@126.com

**Keywords:** UV filter, biomagnification, dietary exposure, aquatic animal, cytochrome P450

## Abstract

2-Ethylhexyl-4-dimethylaminobenzoate (EHDAB) is a commonly used organic ultraviolet filter. The bioaccumulation and biomagnification of EHDAB were investigated in two aquatic animals, the larvae of midge (*Chironomus riparius*) and crucian carp (*Carassius carassius*), and the metabolic enzyme responses in fish liver were determined. EHDAB in the larvae of midge reached a steady state within 10 days of sediment exposure. The biota-sediment accumulation factors ranged from 0.10 to 0.54, and were inversely proportional to the exposure concentrations. The EHDAB-contaminated larvae were used to feed the crucian carp. Within 28 days of feeding exposure, the EHDAB levels in fish tissues gradually increased with the increase of the exposure concentration, exhibiting an apparent concentration-dependence and time-dependence. The liver and kidneys were the main organs of accumulation, and the biomagnification factors of EHDAB ranged from 8.97 to 11.0 and 6.44 to 10.8, respectively. In addition, EHDAB significantly increased the activities of cytochrome P450 (CYP) 1A, CYP3A and glutathione S-transferase in the fish liver. Our results indicate that EHDAB may pose a risk of biomagnification in an aquatic environment and influence the biological processes of exposed organisms.

## 1. Introduction

Organic ultraviolet (UV) filters are important additives to many sunscreens [1]. A large number of organic UV filters have entered into the natural environment due to their extensive use by people [2,3,4,5,6]. Studies have shown that most of the organic UV filters contain monocyclic or polycyclic aromatic structures, possess high hydrophobicity, easily accumulate in sediment as well as different tissues of organisms, and exhibit potential to bioaccumulate in living organisms and to biomagnify in the food web [7,8,9,10]. Considering the ubiquity of organic UV filters in the aquatic environment, increasing attention has been paid to their toxicological effects on aquatic organisms. Recent studies reported that some organic UV filters could influence the growth, development and reproduction as well as induce endocrine disrupting activity in midge, daphnids and fish [11,12,13,14,15,16]. However, the current research has mainly been focused on the investigation of the occurrence in the environment and toxicity testing of organic UV filters. Studies on the trophic transfer and biomagnification of organic UV filters in aquatic ecosystems are still lacking.

2-Ethylhexyl-4-dimethylaminobenzoate is one of the more commonly used organic UV filters, and it has been detected in different water environments, sediments and aquatic organisms. EHDAB was detected in 100% of water samples in Qinhuai River, Jinchuan River, Xuanwu Lake and Yueya Lake within the vicinity of Nanjing City, China, with the concentrations ranging from 3 to 104 ng/L [17]. The maximum concentration of EHDAB reached 110 ng/L in tap water [18]. EHDAB was detected at 150 μg/kg in the sediments of Hong Kong in Victoria Harbour [19]. The highest concentration of EHDAB in marine organisms from Hong Kong coastal waters was 24.1 μg/kg [20]. Since the logarithm of the octanol/water partition coefficient (log*K*_ow_) of this chemical is as high as 6.15 (from Syracuse Research Corporation: http://www.syrres.com/esc/physdemo.htm), EHDAB in water tends to adsorb onto sediments, and the logarithmic values of the organic carbon-normalized distribution coefficients (log*K*_oc_) were obtained and ranged from 4.37 to 4.44 in a lab-scale water-sediment system set up with natural water and sediment samples collected from Nanjing, East China [21]. EHDAB can also be found to concentrate in fish by waterborne exposure and induce oxidative stress [22].

In the present paper, we investigated the bioaccumulation potential of EHDAB in the larvae of midge (*Chironomus riparius*) by sediment exposure, explored the biomagnification potential of EHDAB in crucian carp (*Carassius carassius*) by feeding them live EHDAB-contaminated larvae in a lab-scale aquatic environment, and determined the metabolic enzyme responses induced by EHDAB in fish liver. This study will be very valuable for the assessment of the potential risk from EHDAB in aquatic ecosystems.

## 2. Materials and Methods

### 2.1. Reagents and Materials

EHDAB (CAS No. 21245-02-3, purity > 98.5%) was purchased from Sigma-Aldrich (Flanders, NJ, USA). 7-Ethoxyresorufin, resorufin, resorufin pentyl ether, 7-benzyloxy-4-trifluoromethyl coumarin (BFC), 7-OH-4-trifluoromethyl coumarin and tricaine methane sulfonate (MS-222) were purchased from J & K Scientific (Shanghai, China). Methanol, dichloromethane, and ethyl acetate (HPLC grade) were purchased from the Merck Serono Co., Ltd. (Darmstadt, Germany). Kits for the analysis of glutathione S-transferase (GST) and the protein level were supplied by the Nanjing Jiancheng Bioengineering Institute (Nanjing, China). Oasis hydrophilic/lipophilic balanced (HLB; 6 cc, 200 mg) solid-phase extraction cartridges were purchased from Waters (Milford, MA, USA).

### 2.2. Testing Organisms and Exposure

Fourth instar larvae of midge (2–4 cm body length) were obtained from an aquarium shop in Nanjing and were acclimatized in culture medium (0.5 mM CaCl_2_, 1 mM NaCl, 1 mM MgSO_4_, 0.1 mM NaHCO_3_, 0.025 mM KH_2_PO_4_, 0.01 mM FeCl_3_) supplemented with wheat bran at 21 ± 2 °C with a light/dark photoperiod of 16 h/8 h for 7 days. Natural sediment samples were collected from Qinhuai River in Nanjing, China. Freeze-dried sediment samples, deionized water and glass instruments were autoclaved for 20 min at 121 °C and 1.3 bar before exposure to prevent the biodegradation of EHDAB. Based on the reported EHDAB contents in the sediments in Hong Kong and Japan [19], 800 mL of EHDAB solutions with nominal concentrations of 4, 20, 100 and 500 μg/L were mixed with 800 g of the sterilized sediments and spread in a 4-L glass tank to form a sediment layer of about 2 cm. After two hours for stabilization, around 5 g wet weight of larvae (150–180 individuals) were placed into the container. A blank control (deionized water and sediments) and a solvent control (deionized water containing 0.01% methanol and sediments) were included. All of the treatments were replicated three times simultaneously. The larvae (about 30 individuals) and sediments were collected for EHDAB quantitation at 0, 2, 4, 7 and 10 days of exposure, and the larval survival was recorded. No food was provided during the exposure period.

Juvenile crucian carp (about eight months old, 30 ± 10 g, 10 ± 2 cm) were obtained from the Nanjing Institute of Fishery Science (Nanjing, China). All fish were acclimatized in dechlorinated municipal water and fed with unexposed midge larvae twice daily. Faeces and uneaten food were removed every day by suction. Twelve fish of either sex were kept in 50 L glass tanks randomly containing 30 L of dechlorinated tap water under constant aeration. Three dietary intake levels of EHDAB along with an uncontaminated control were tested. The midge larvae exposed to 20, 100 and 500 μg/L of EHDAB for 10 days were used as bait in the process of exposing the fish through feeding. The fish were fed once each day at a daily rate of 3% of the total weight of the fish. During the exposure period, half of the water was replaced with fresh dechlorinated tap water every day. All treatments were performed in triplicate. The animal care and experimental protocols followed the standard procedures of the Institutional Animal Care and Use Committee.

For EHDAB quantitation, two fish of every replicate experiment were pooled at 7, 14 and 28 days of exposure, and the liver, brain, gill, kidney, skin and muscle tissues were collected. For enzyme assays, two fish were sacrificed from each replicate tank at 3, 7 and 14 days of exposure, and the liver was immediately removed. Fish were anaesthetized with MS222 (100 mg/L) and killed by cervical transection. All tissue samples were washed with 0.15 M KCl, dried in filter paper, and then immediately frozen in liquid nitrogen.

### 2.3. Sample Extraction and Chemical Analysis 

Sediment and tissue samples were extracted by means of pressurized liquid extraction (ASE 350, Dionex, Sunnyvale, CA USA). The detailed procedure has been described in our previous study [21,22]. Freeze-dried samples (~2 g for sediment, ~0.5 g for liver and ~1 g for the other tissues) were thoroughly mixed with diatomite. The mixture was then added into a 22 mL stainless steel extraction cell containing glass fibre filters in both the inlet and outlet of the cell. A mixture of ethyl acetate/n-hexane (80/20, volume/volume (*v*/*v*)) was used as the extraction solvent. The extracts of sediments were passed through an Oasis HLB solid-phase extraction cartridge at a flow rate of 5 mL/min, which was preconditioned with dichloromethane (6 mL), methanol (6 mL) and ultrapure water (6 mL). And then, the cartridge was washed with 10 mL ultrapure water and dried under vacuum for 0.5 h. The cartridge was then eluted using 2 × 5 mL methanol/dichloromethane (1:1, *v*/*v*), and the eluates were evaporated, reconstituted with methanol, and stored at −20 °C until further analysis. The extracts of tissues were also evaporated under a stream of nitrogen, reconstituted with 1 mL of methanol, and then centrifuged for 15 min (12,000× *g*) at 0 °C to remove the lipids. Approximately 0.5 mL of the supernatant fluid was removed and preserved at −20 °C until further analysis.

EHDAB concentrations were determined using ultra-high performance liquid chromatography coupled with tandem mass spectrometry (UPLC/MS/MS, Agilent, Waldbronn, Germany). Separation was performed on an Eclipse Plus C18 (2.1 × 30 mm, 1.7 μm) column. Detection was performed with a positive ion electrospray ionization source (ESI). The ions were monitored by the multiple-reaction monitoring (MRM) mode. Matrix recoveries were determined by triplicate analyses of sediment and biota samples spiked with EHDAB before pre-process. The limits of detection (LODs) and limits of quantitation (LOQs) of EHDAB in the sediment samples were 5.2 × 10^−3^ and 1.7 × 10^−2^ ng/g dry weight (dw), and the recovery was 78%. The LODs and LOQs of EHDAB in the biota samples were 0.2–0.4 and 0.8–2 ng/g lipid weight (lw), respectively, and the recoveries were 81–107%. More specific details can be found in Ma et al. [22].

The subtraction method was used to determine the lipid contents (wet weight) in different fish tissues and midge larvae [23]. The organic carbon content was determined by a Total Organic Carbon (TOC) analyser (Model 1030, OI Analytical, College Station, TX USA).

### 2.4. Determination of Enzyme Activities

Liver samples were homogenized in a 1:9 (weight/volume) Tris-HCl buffer solution (0.1 M Tris-HCl, pH = 7.4, 0.15 M KCl) and centrifuged for 30 min at 9000× *g* at 4 °C. The supernatants were used as the extract for the analysis of the enzymatic activities. A microplate reader (Synergy H4, BioTek, Winooski, VT USA) was used for carrying out the measurements. The GST activity was determined at 340 nm using 10 µL of homogenate and 170 µL of the reaction solution according to the method of Frasco and Guilhermino [24]. The catalytic activities of 7-ethoxyresorufin-O-deethylase (EROD), 7-penthoxyresorufin-O-deethylase (PROD) and 7-benzyloxy-4-trifluoromethylcoumarin-O-debenzylase (BFCOD) were measured and determined as the conversion rates of 7-ethoxyresorufin to resorufin, resorufin pentyl ether to resorufin, and BFC to 7-OH-4-trifluoromethylcoumarin, respectively, in 96-well microplates [25,26]. The reaction was monitored for 30 min by measuring the fluorescence every 10 min at excitation and emissions wavelengths of 530 nm and 590 nm for EROD and PROD along with 410 nm and 538 nm for BFCOD. Protein concentrations in the liver were determined using the method developed by Bradford [27] with bovine serum albumin as the standard.

### 2.5. Statistical Analysis

All data were tested for a normal distribution by the Shapiro-Wilk’s test and the homogeneity of variances by Levene’s test. Data from different treatments were analyzed by multifactor ANOVA followed by Duncan test. All data were expressed as the mean ± standard deviation, and *p* < 0.05 was considered statistically significant. Statistical analysis was processed by the SPSS statistical package (ver. 21.0, SPSS Inc., Chicago, IL, USA).

## 3. Results and Discussion

### 3.1. Bioaccumulation of EHDAB in Midge Larvae

There were no significant changes in the average weight for the chosen midge larvae during the entire exposure periods. No EHDAB was detected in sediments or larvae in the blank and solvent controls. The measured concentrations of EHDAB in sediments are shown in Table 1, and C1, C2, C3 and C4 represent the experimental groups with nominal exposure concentrations of 4, 20, 100 and 500 μg/L, respectively. The concentrations of EHDAB decreased during the 10 days exposure period in all of the treatment groups. The reasons may be the accumulation of midge larvae and the photodegradation of EHDAB [28,29]. However, all the decreases are not statistically significant at the 95% confidence level. The concentrations of EHDAB in each group were not less than initial concentration of 85% after 10 days of exposure.

**Figure 1:**
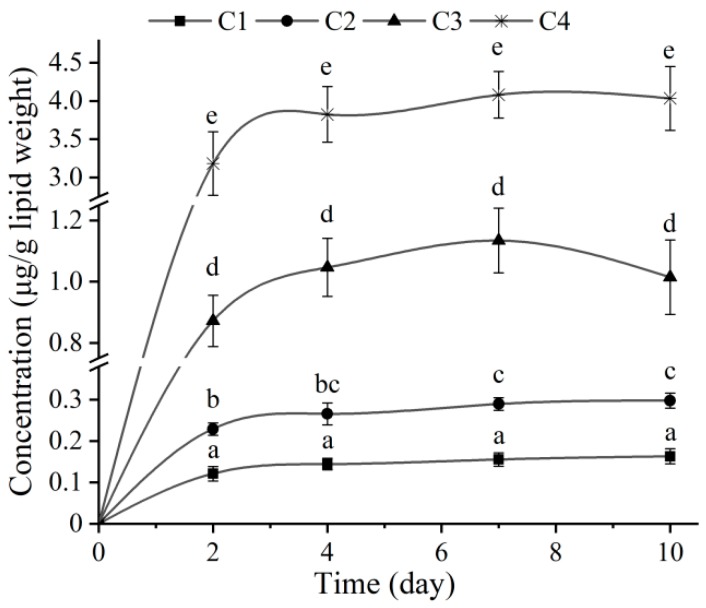
Concentration changes of EHDAB in larvae during 10 days of exposure. C1, C2, C3 and C4 represent the experimental groups with nominal exposure concentrations of 4, 20, 100 and 500 μg/L, respectively. Values are expressed as the mean ± standard deviation (*n* = 3), and different letters above the bars indicate significant differences between treatments and among time for a given treatment at the *p* < 0.05 level between treatments.

The survival rates of larvae were 100% after 10 days of exposure, and the changes in the EHDAB concentrations in larvae in different treatments with time are shown in Figure 1. The EHDAB concentrations in the larvae significantly increased with increasing exposure concentrations (*p* < 0.05). Regarding the time responses, the EHDAB concentrations in the larvae gradually increased in the first 7 days of exposure for all the treatments. The rising trend was sharp during the first two days and then slowed down from 2 to 7 days. From 7 to 10 days, the concentrations of EHDAB in the larvae in the two lower concentration groups (C1 and C2) still slightly increased, while slightly decreased in the C3 and C4. In general, the EHDAB concentrations in the larvae did not significantly increase after day 2, except for the C2 treatment group, and EHDAB seemed to reach a steady state after 10 days of exposure.

To estimate the bioaccumulative effect of midge larvae on EHDAB in sediment, the biota-sediment accumulation factor (BSAF) was calculated dividing C_org_ (the lipid-normalized concentration in larvae, ng/g lipid) by C_s,oc_ (the organic carbon normalized concentration of EHDAB in sediment, ng/g oc). The organic carbon content in the sediment used for the exposure of midge larvae was determined to be 1.1% with a TOC analyser. The BSAF values of EHDAB were 0.54 and 0.19 for C1 and C2 at 10 days, while they were 0.14 and 0.10 for C3 and C4 at 7 days, respectively, suggesting an inverse relationship to the exposure concentration. A previous study also showed that the measured BSAF values of phenanthrene, pyrene and chrysene in *Chironomus plumosus* larvae were all lower than 1.0, and the authors suggested that these labile PAHs could be metabolized by *C. plumosus* larvae [23]. In our research, EHDAB tends to have lower BSAF values (<1.0) might because EHDAB possesses a stronger sorption affinity in sediments which results in lower bioavailability. Besides, the midge larvae used in this study may have the ability to metabolize EHDAB. The BSAF values of EHDAB decreased with increasing exposure concentrations, which may be due to the tissue saturation caused by the high exposure concentrations. This result is consistent with a previous study on the bioaccumulation of ibuprofen in mussels [30].

### 3.2. Biomagnification of EHDAB in Crucian Carp

**Figure 2:**
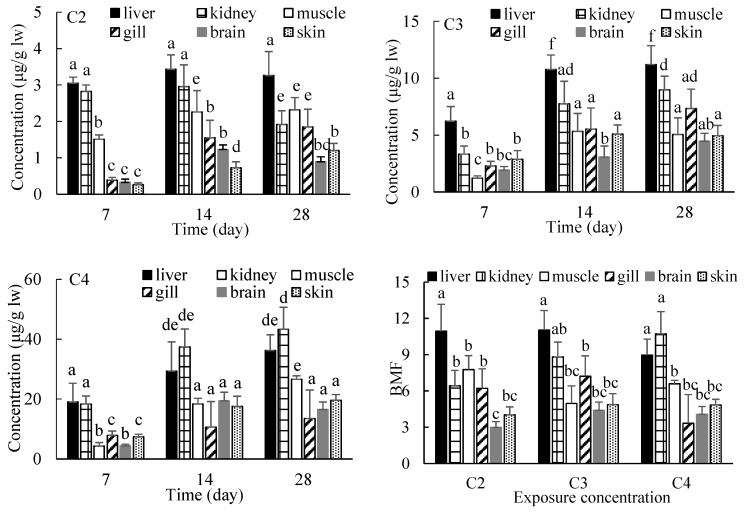
Concentrations and BMFs of EHDAB in different fish tissues by feeding exposure (C2, 0.2977 μg/g lw; C3, 1.014 μg/g lw; and C4, 4.033 μg/g lw). Values are expressed as the mean ± standard deviation (*n* = 3), and different letters above the bars indicate significant differences at the *p* < 0.05 level between treatments.

According to the results of the bioaccumulation of EHDAB in midge larvae, the larvae exposed to the C2, C3 and C4 groups for 10 days were used to feed crucian carp for assessing the biomagnification effects of EHDAB. Due to the very short contact time with water, the loss of the compound from midge larvae into the water phase during feeding was minimized, making it possible to link the accumulation and effects in the fish directly to the dietary intake levels. No EHDAB was detected in fish in the controls or in the water in all the treatments during the exposure period of 28 days. Changes in the EHDAB concentrations with time in different tissues are shown in Figure 2.

The accumulation of EHDAB in fish tissues showed apparent dependencies on the concentration and time, and significant differences were found for the tissue distribution in all cases. In general, the increase of EHDAB levels in fish tissues was correlated with the increase of the exposure concentration. The concentrations of EHDAB in the liver and kidneys were significantly higher than those in the other tissues in most cases (*p* < 0.05). Besides the liver and kidneys, the muscle was also a main accumulative tissue in the C2 treatment group, while the accumulation amounts were relatively low in the brain and skin. At the end of the exposure period, there were no significant differences between the brain and skin tissue for different concentrations.

With regards to the time responses, the EHDAB concentrations in all of the tissues significantly increased from day 7 to day 14, except for the liver and kidneys at C2 group and the brain at C3 group. However, from day 14 to day 28, the EHDAB concentrations did not changed significantly for most of the tissues regarding different exposure concentrations, and significant increases were observed only in the gills and skin at C2 group and the muscle at C4 group. It suggested that the accumulation of EHDAB in fish basically attained steady-state within 28 days.

The present results showed that the liver is main organ of accumulation with feeding exposure, which is consistent with previous studies on antifungal medication and UV filters with waterborne exposure [22,31]. The reason might be attributable to the role of this organ as the primary site for the metabolism of xenobiotics. The kidney is an important organ for the excretion of pollutants, and when EHDAB could not be effectively transformed in the liver, this likely resulted in the concentration increasing in the kidneys [32]. A previous study reported the preferential accumulation of erythromycin in the gills of crucian carp following waterborne exposure [33]. Furthermore, the position of the gills between the venous and arterial circulation allows the accumulation of chemicals on them, which were absorbed by another route of exposure as food intake [34].

Biomagnification factor (BMF) values were calculated at day 28 by dividing the EHDAB concentration in the crucian carp tissues by the EHDAB concentration in the larvae of midge after normalizing the values for the respective lipid contents. BMF values exhibited tissue variability (Figure 2), the largest BMF values were obtained in the liver (8.97–11.0) and kidneys (6.44–10.8), while smallest in the brain (2.99–4.08). However, the concentration dependence was unapparent for BMF in most tissues.

In a field study, the BMF value of the UV filter 2-ethyl-hexyl-4-trimethoxycinnamate in different trophic levels in the Swiss lake was 1.5 [35]. Compounds are considered to have the risk of biomagnification along the food chain when the BMF value is greater than 1. The BMF values of EHDAB in different fish tissues in this study were all greater than 1, indicating that EHDAB possesses the risk of biomagnification in the actual aquatic environment.

### 3.3. Effects of EHDAB on Metabolic Enzymes in Fish

**Figure 3:**
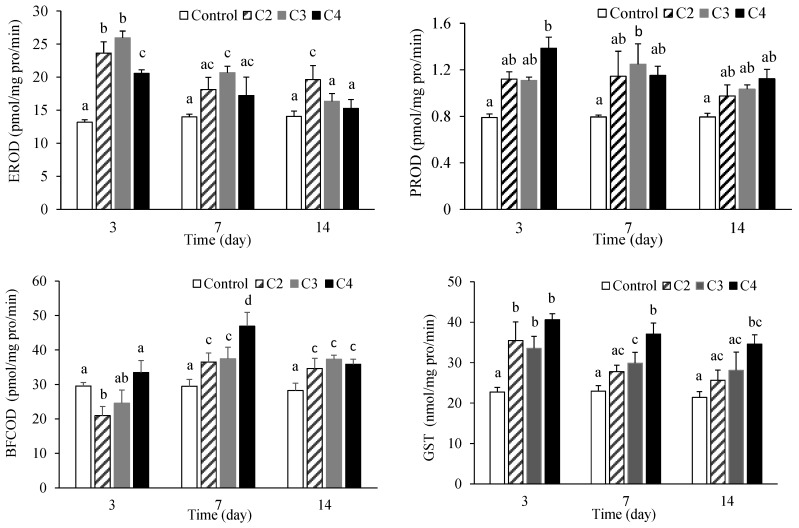
Responses of metabolic enzymes in fish upon dietary exposure to EHDAB (C2, 0.2977 μg/g lw; C3, 1.014 μg/g lw; and C4, 4.033 μg/g lw). Values are expressed as the mean ± standard deviation (*n* = 3), and different letters above the bars indicate significant differences at the *p* < 0.05 level between treatments.

Changes in the EROD, PROD, BFCOD and GST activities in the fish liver after exposure to EHDAB are presented in Figure 3. The EROD activity significantly increased for all the exposure groups at day 3 relative to the activities in the controls, although the induction of EROD was reduced at the highest concentration. However, after 7 and 14 days of exposure, the activity of EROD was significantly increased only by the C3 treatment at day 7 and C2 treatment at day 14. No obvious differences were observed between the other treatments and the controls, and the induction of EROD activity showed a tendency to decrease with increased exposure time. The biggest induction was almost 2-fold for the C3 treatment at day 3.

The dietary exposure of EHDAB increased the PROD activity in all cases, and the PROD activity significantly increased 0.58-fold for the C3 treatment at day 7 and 0.75-fold for the C4 treatment at day 3 (*p* < 0.05). The BFCOD activity increased in all cases with the exception of the two lower concentrations at day 3. EHDAB significantly increased the BFCOD activity at days 7 and 14 for all of the exposure concentrations (*p* < 0.05), and the biggest increase was observed for the C4 treatment at day 7, with a 0.59-fold increase.

Comparing the activity values of the three P450 enzymes and their degrees of responsiveness to EHDAB exposure, the EROD activity is the most sensitive indicator, with a rapid increase at day 3. The induction of BFCOD activity was lagging behind that of EROD. However, the PROD activity in fish liver was much lower than those of EROD and BFCOD in either controls or treatments, and EHDAB exposure did not significantly increase the PROD activity in most cases.

The cytochrome P450 enzymes (CYP450s) are involved in the metabolism of pharmaceuticals and other xenobiotics, in which CYP1, CYP2, and CYP3 are the three main families [36]. The activity of CYP1A is traditionally measured as the catalytic activity of EROD, and the EROD activity is widely used as an indicator of environmental contamination. Many pharmaceuticals, such as roxithromycin, propranolol and diphenhydramine, etc., were reported to induce EROD activity in fish liver [37,38,39]. The induction of CYP1A is generally mediated via the aryl hydrocarbon receptor (AhR) pathway [40]. In the present work, significant induction of EROD was observed in the fish liver, which suggested a possible interaction between EHDAB and AhR.

CYP3A is another major CYP responsible for the metabolism of drugs, and the induction of CYP3A enzyme activity is generally measured by BFCOD [36]. In a previous study, 100 μg/g of roxithromycin acutely increased BFCOD activity and induced CYP3A mRNA expression in the liver of crucian carp after 7 days of exposure [41].

There is currently a lack of information about the effects of xenobiotics on the CYP2B-associated PROD in fish species in the literature. The EROD and PROD activities in rainbow trout (*Oncorhynchus mykiss*) significantly increased after 21 days of exposure to the UV filter 2-phenylbenzimidazole-5-sulfonicacid at concentrations of 1, 10 and 1000 μg/L; however, no statistically significant increases in BFCOD activity were found among the control and treatments [42].

Overall, the present study revealed that EHDAB can alter the EROD, PROD and BFCOD activities. However, CYP induction may not mean that these enzymes actually metabolized EHDAB. Previous studies reported that some chemicals (e.g., co-planar polychlorinated biphenyls) induced CYPs but were not significantly metabolized [43,44]. Recent studies identified EHDAB metabolites in human urine following its application on skin [45] as well as in rabbit liver and kidneys after intravenous administration [46]. Unfortunately, information on EHDAB metabolism in fish is unavailable. Therefore, further research will be needed to identify the metabolites of EHDAB in fish.

The GST activity significantly increased by dietary exposure to EHDAB in all of the treatments with the exception of the lowest concentration at days 7 and 14. The GST induction slightly decreased along with increased exposure time. In general, the GST activity showed a concentration-dependent increase.

The critical toxicity of any specific xenobiotic that is metabolized through both routes I and II depends on the capacity of the phase II enzymes to conjugate the reactive metabolites produced by phase I biotransformation reactions [24]. GST is an important enzyme in organisms for phase II metabolic reactions, as it can catalyse a variety of electrophilic compounds combined with glutathione and promote glutathione’s removal from the organism [47]. The elevated levels of GST were usually observed together with EROD increases in fish livers exposed to pharmaceuticals, such as roxithromycin, erythromycin and diclofenac [33,37,48]. These are consistent with our results, suggesting that metabolism and excretion of these substances might take place in fish.

## 4. Conclusions

The present study showed that the larvae of midge can intake EHDAB from sediment, and the levels of EHDAB increased with increases in the exposure concentration and time, although the BSAF values were always less than 1. EHDAB was transferred from the larvae of midge to the fish, and the maximum concentrations were detected in the fish liver and kidneys in most cases. BMF values exhibited concentration-dependence and tissue-specificity, ranging from 3 to 11 after 28 days of dietary exposure. EHDAB in fish resulted in a significant induction of phase I and II metabolic enzymes, and EROD, BFCOD and GST activities in the liver seemed to be more sensitive indicators. However, further research is still required to confirm the biomagnification potential of EHDAB in aquatic environments as well as whether the alterations of the activities of CYPs and GST are related to the accumulation and metabolism of EHDAB.

## Figures and Tables

**Table 1:** The measured concentrations of EHDAB in sediments with different treatments.

Concentration (ng/g dw)	0 d	2 d	4 d	7 d	10 d
C1	3.9 ± 0.7	3.9 ± 0.8	3.8 ± 0.6	3.6 ± 0.7	3.3 ± 0.5
C2	19.2 ± 3.6	18.8 ± 2.8	18.7 ± 2.3	18.1 ± 1.9	17.2 ± 2.1
C3	95.6 ± 12.8	94.7 ± 11.5	92.5 ± 10.6	89.5 ± 11.9	84.6 ± 9.4
C4	486.6 ± 24.2	482.1 ± 37.8	479.2 ± 28.2	467.7 ± 36.4	445.5 ± 32.9
